# Assessment of provably secure hybrid authenticated key exchange for dual-use applications

**DOI:** 10.1140/epjqt/s40507-026-00532-9

**Published:** 2026-06-11

**Authors:** Simon Abelard, Juan Antonio Vieira Giestinhas, Delaram Kahrobaei, Ludovic Perret, Timothy Spiller, Christoph Striecks

**Affiliations:** 1 LRE, EPITA, 14 rue Voltaire, Le Kremlin Bicêtre, 94270 France; 2 LIP6, Sorbonne Université, 4 Place Jussieu, Paris, 75005 France; 3 School of Physics, Engineering and Technology, University of York, Information Centre Market Square, York, YO10 5DD UK; 4 PhD Program in Mathematics, CUNY Graduate Center, 365 5th Ave, New York, NY 10016 USA; 5 Departments of Computer Science and Mathematics, CUNY Queens College, New York, USA; 6 Department of Computer Science, New York University, New York, USA; 7 AIT Austrian Institute of Technology GmbH, Giefinggasse 4, Vienna, 1210 Austria

**Keywords:** Post-Quantum Cryptography (PQC), Quantum Key Distribution (QKD), Hybrid Authenticated Key Exchange (HAKE), Dual-use applications

## Abstract

This work assesses the literature on QKD-PQC Hybrid Authenticated Key Exchange (HAKE) and related schemes, based on security proofs (e.g. availability of such a proof and underlying model), performance, and near-term implementation. We evaluate these protocols across diverse dual-use environments, ranging from strategic military SATCOM to high-value civilian infrastructure, such as banking backbones. The trade-offs among various solutions are discussed based on their Technology Readiness Level (TRL) and performance. Moreover, we identify specific use cases such as untrusted satellite nodes or long-distance terrestrial links which might require alternative measures depending on the operational requirements.

## Introduction

Quantum technologies are challenging today’s digital security [9]. Shor’s algorithm [84] combined with potential emergence of a sufficiently large quantum computer will make obsolete the first public-key cryptography standards, such as RSA (Rivest-Shamir-Adleman), Diffie-Hellman (DH), Elliptic Curve DH (ECDH) or Elliptic Curve Digital Signature Algorithm (ECDSA), which form the backbone of current digital security.

A new generation of cryptography, known as quantum-safe cryptography, provides two complementary approaches to circumvent this quantum threat: post-quantum cryptography (PQC) [14] and quantum cryptography [42], in particular, Quantum Key Distribution (QKD) [12].

The security of QKD holds under the assumption that the laws of quantum physics are valid. More precisely, a central concept is the no-cloning theorem, which states that it is impossible to create an identical copy of an arbitrary unknown quantum state using a unitary operation: an unconditional eavesdropper cannot clone the quantum state without disturbing it, thereby revealing their presence [12, 42, 80].

In contrast, the security of PQC relies on the computational hardness, both in the classical and quantum settings, of new algorithmic problems. In particular, the US National Institute of Standards and Technology (NIST) released^1^ in 2024 a first set of Federal Information Processing Standards (FIPS) for public-key PQC. This release establishes the post-quantum requirements for two core functions: Key Encapsulation Mechanisms (KEM), which secure the exchange of encryption keys, and Digital Signature Algorithms (DSA), which verify data integrity and authenticity. Specifically, the set consists of ML-KEM (FIPS 203) and ML-DSA (FIPS 204), both based on Module-Lattice (ML) problems, as well as SLH-DSA (FIPS 205), which utilizes Stateless Hash-based (SLH) structures. Additional algorithms with different underlying security assumptions were also selected: Falcon, a DSA based on a structured lattice, and HQC (Hamming Quasi-Cyclic), a KEM based on error-correcting codes, whose FIPS specifications are underway.

While QKD provides the highest theoretical security, namely information-theoretical security (ITS), current practical implementations are typically limited in terms of distance between nodes [98]. To extend QKD networks over longer distances, intermediate nodes are introduced to relay the signal. These trusted nodes must be physically secure, as they handle the key material during transmission. These nodes preclude the possibility of end-to-end quantum security, which may be questionable on both operational and ethical grounds. It is also important to note that QKD requires a classical authentication mechanism, which can also be made ITS with message authentication codes (MAC) based on universal hash functions [77, 94]. However, this increase of security comes with an increase of key consumption, since the ITS MAC functions require a fresh key every time they are used to preserve the ITS security.

Until now, it has been common to oppose QKD and PQC, e.g. [19, 79]. One reason is that PQC is by definition incapable of providing information-theoretic security (i.e. resilience against a computationally unbounded attacker), so one could argue that PQC would bring little to a system relying on QKD. Furthermore, the lack of maturity of new PQC hard problems lays ground for criticism as two of the most promising candidates to the NIST competition, Rainbow [15] and SIKE [23], were broken using classical means (a simple laptop in both cases). The relative novelty of post-quantum schemes also means more opportunities for side-channel attacks, as can be seen by the significant increase of papers published in that field.

On the other hand, QKD has been vastly criticized too for its practical limitations [80]: the lack of authentication, the vulnerability to Denial-of-Service (DoS) attacks, the difficulty to produce cost-efficient and reliable hardware, and the distance limitation which makes QKD incapable of providing end-to-end security on a sizable network.

Our approach is more holistic, and we prefer to study quantum-safe communications in a broader sense, taking into account any cryptographic mechanism that is unaffected by the advent of quantum computers. This includes QKD, symmetric and post-quantum public-key cryptography, technologies capable of complementing each other.

In this context, it makes sense, particularly, for sensitive applications, to combine both quantum and post-quantum cryptography as these are both quantum-safe and rely on different security assumptions. In the unfortunate event that PQC and QKD are rendered insecure, classical material can also be included using the same cryptographic functions used to combine PQC and QKD. We emphasize that this should not be seen as distrusting either quantum or post-quantum cryptography, but rather as a conservative approach to ensure robustness and crypto-agile systems, where the security of the session remains intact as long as at least one of the underlying primitives remains secure.

### Contribution

In this paper, we review the various techniques to perform secure hybridization of post-quantum and quantum cryptography, with a focus on higher Technology-Readiness-Level (TRL) and proven-secure solutions. For instance, this works covers a substantial variety of QKD protocols and the existing PQC algorithms to analyze their performance and suitability in terrestrial and satellite based infrastructures, while commenting the current deployment to analyze their dual-use applications. Satellite-based applications are of particular interest, as QKD offers the potential to achieve significantly greater key-exchange distances than for terrestrial nodes, typically connected via optical fibers. Once the QKD and PQC aspects are commented, various hybrid options from the literature are analyzed with the following criteria: security, feasibility in the short run, and performance (e.g. key rate). This analysis first allows us to narrow down our list to a limited number of choices that are both feasible and secure, and to provide guidelines on which of the remaining options to prefer depending on the context. We hope that this analysis can be of interest beyond the scientific community and serve as a basis to help engineers and Standards Developing Organizations (SDOs) in their work.

More precisely, our conclusion is that high security applications should be using what we call “Combined-Hybrid Protocols”, in which quantum and post-quantum parts are carefully combined in a *provably secure way*. As we will see in Sect. 4, the hybrid AKE protocols from Table 1 are currently valid options depending on the concrete use case. To instantiate the QKD part, the most suitable building blocks are: Decoy-States BB84 [12] for satellite-based QKD where it is acceptable to trust the satellite (e.g. for military applications), or fiber-links between critical infrastructure such as finance institutions, and source-independent protocols such as BBM92 [13] when the satellite cannot be trusted and lower key rates are acceptable. See Sect. 5 for discussions on this matter based on the use cases that we consider.

**Table 1:** Overview of HAKE and related protocols with security proofs. ITS refers to information-theoretic security where no restrictions are based on the power of the adversary while computational adversaries are bounded. For everlasting security, we require the adversary to be computationally bounded until the end of the protocol run only; afterwards there are no restrictions on the adversary. NBB stands for non-black-box

Protocol	Model	Security	Comment
Muckle [30]	HAKE	Comput.	Uses pre-shared keys (PSKs) for authentication
Muckle+ [18]	HAKE	Comput.	Uses post-quantum signatures for authentication
Muckle++ [41]	HAKE	Comput.	ITS primitive for PRF, but with HKDF
Muckle# [10]	HAKE	Comput.	Uses a post-quantum KEM for authentication
VMuckle [21]	HAKE	Comput.	Adds flexible options (PSK or signature)
ITS AKE [47]	CK^+^	ITS	Assumes ITS-authenticated QKD channel
NBB AKE [38]	NBB	Comput.	QKD is not a black box
VMuckle-ES [36]	HAKE	Everlasting	Mix of computational and ITS guarantees

### Background

The focus of this work is on so-called Hybrid Authenticated Key Exchange (HAKE) and related protocols, which combine classical, post-quantum, and quantum components, with a security proof. In particular, Dowling, Hansen, and Paterson [30] introduced a popular security framework for HAKE that allows proving security properties, such as forward security, post-compromise security, and resilience in the event of component failures.

This security framework was adopted by a series of new hybrid protocols [10, 18, 21, 30, 41], dubbed Muckle, Muckle+, Muckle++, Muckle# and VMuckle, proven in such HAKE security framework and that mostly differ on the method used for authenticating the parties. VMuckle slightly differs from the other protocols in that it has been designed to be modular on the authentication technique used, aiming for deployment in a particular security protocol MacSec.

In [47], the authors introduced a new security framework, based on CK^+^ [46] allowing to capture additional security features, notably depend-key attacks (but no post-compromise security), and proved their scheme information-theoretically secure in the CK^+^ framework.

All the results mentioned before treated QKD as a black-box with perfect security compared to the other cryptographic components. Practical deployments of QKD demonstrated that reality is more subtle. The work in [38] proposed a new security model and a new hybrid protocol to take into account the specificity of QKD.^2^

In Table 1, we summarize the HAKE and related protocols and regroup their main features. A more detailed table is presented in [47], with more focus on security guarantees and including protocols that are not all AKE protocols. We emphasize that our study is focused on provably-secure AKE, leading us to exclude works such as [40, 81], where neither an AKE nor a security proof is provided. As for the underlying QKD protocols and PQC algorithms, see Table 2 and Table 3, respectively, for more details on the list of potential candidates to be implemented.

**Table 2:** Technical landscape of representative QKD protocols. This non-exhaustive selection highlights protocols commonly established in literature or industry. Note that categories such as CV, DV-MDI, and TF represent broad families; values provided reflect typical performance for their standard implementations

Protocol	Device independence	TRL	Secret bits/pulse (SB/P)			Comment
			20 dB	40 dB	80 dB	
BB84	None	9	≳10^−4^	10^−5^	N/A	Commercial fiber choice
SSP	None	5-7	10^−4^	10^−7^	N/A	Finite-size effect not analyzed
SARG04	None	5-7	10^−7^	N/A	N/A	Alternative for Decoy-States
RFI	None	5-7	≳10^−4^	10^−6^	N/A	Potential candidate for satellite
DPS	None	5-7	10^−4^	N/A	N/A	Recently proven ITS with finite size
RRDPS	None	5-7	≲10^−4^	N/A	N/A	Operates at any QBER below 50%
COW	None	7-9	≳10^−8^	N/A	N/A	Recently proven ITS with finite sizen
DI	Source & Det.	3-4	N/A	N/A	N/A	Low loss only
BBM92	Source	5-7	10^−5^	10^−8^	N/A	Untrusted satellite use
DV MDI	Detectors	5-7	≲10^−4^	10^−7^	N/A	Good for star topologies
TF	Detectors	5-6	≳10^−4^	≲10^−4^	10^−7^	Feasible for high loss (>120 dB)
MP	Detectors	5-6	10^−4^	10^−5^	≳10^−9^	Less complex than TF to deploy
SCS	Detectors	4-6	≲10^−9^	N/A	N/A	TF variant with imperfected sources
CV	None	7-8	≲10^−3^	10^−7^	N/A	High performance for low loss

**Table 3:** Standardized and to-be-standardized PQC algorithms by NIST

Standard	Primitive	Hardness assumption
SP 800-208 (XMSS and LMS)	DSA	Preimage and collision resistance of hash function
FIPS 203 (ML-KEM)	KEM	Module Learning with Errors (MLWE)
FIPS 204 (ML-DSA)	DSA	Module-LWE and Short Integer Solution (MSIS)
FIPS 205 (SLH-DSA)	DSA	Preimage and collision resistance of hash function
FIPS 206 (FN-DSA)	DSA	Short Integer Solution (SIS) over NTRU Lattices
FIPS 207 (HQC)	KEM	Quasi-Cyclic Syndrome Decoding

### Outline

This work begins with a review of current QKD protocols in Sect. 3. The presented protocols are evaluated based on TRL and performance metrics, specifically secret bits per pulse, to assist readers in selection, while omitting an exhaustive analysis of implementation costs. Furthermore, the global deployment of QKD is discussed to highlight dual-use applications that extend to the hybrid key exchange scenario. Section 4 shortly introduces the relevant PQC algorithms before exploring the hybrid integration of both technologies; this combination forms the basis of the HAKE and related protocols, which are analyzed and compared based on their security guarantees. Finally, Sect. 5 presents realistic, short-term use cases for the reviewed protocols.

## Declaration

### Ethical approval

This work is purely theoretical and did not require ethical approval from any of the respective institutions.

As this work evaluates the suitability of hybrid authenticated key exchange protocols for dual-use applications, we acknowledge that two authors of this work (L. Perret and C. Striecks) have also co-authored a subset of the protocols we review (namely, Muckle+ [18], Muckle# [10], and VMuckle [21]). Moreover, C. Striecks is also involved in the ETSI technical specification of VMuckle-ES [36].

### Acknowledgments

We conducted this work partially with the support of ONR Grant 62909-24-1-2002. Moreover, this work received funding from the European Union’s Horizon Europe research and innovation program under agreement number 101114043 (“QSNP”). JAVG has conducted this work with the support of EPSRC PhD studentship Grant EP/W524657/1.

## Quantum key distribution

Quantum cryptography utilizes the properties of quantum mechanics to develop primitives that would otherwise be impossible to achieve. Whilst the field encompasses a wide variety of protocols, e.g. Quantum Secure Direct Communication [92] and Blind Quantum Computing [39], these vary in feasibility. For example, Quantum Key Distribution (QKD) is notable for its high maturity and successful real-world implementation. Considering that QKD is both the easiest to combine with PQC and the most mature solution in terms of TRL, this work focuses on the hybridization of QKD and PQC.

### QKD taxonomy and fundamental transmission constraints

QKD protocols come in numerous flavors, based on the following characteristics: whether they use *continuous* or *discrete* variables (CV or DV QKD), the trust level they place in Alice and Bob’s devices from device-dependent (DD) to Measurement-Device-Independent (MDI-QKD), Source-Independent (SI-QKD) and fully device-independent (DI), the type of protocol used: *Prepare-and-measure* (PM) or *Entanglement-based* (EB) and the technology used to realize the quantum link: single-photon sources (SPS), weak coherent pulses (WCP), etc. Despite the wide variety of QKD protocols, their security is always based on the laws of physics, notably on quantum mechanics and the derived tools such as the no-cloning theorem. Quantum information tools are used to determine mutual information between honest parties and unconditional eavesdroppers, required to estimate the final secure secret key rate of the QKD protocols.

While traditional QKD protocols assume perfectly characterized hardware, any deviation between an implementation and the assumptions used for its theoretical security proof, often caused by imperfect devices, can introduce vulnerabilities. MDI-QKD (SI-QKD) protocols mitigate this by relying on the laws of quantum mechanics, which allows the measurement devices (source) to be imperfect, effectively making the central node or relay untrusted. In contrast, DI-QKD offers the highest security posture by providing immunity to all side-channel attacks and hardware imperfections, as its security is verified through the violation of the Bell inequality rather than hardware characterization [6, 102].

A fundamental challenge in QKD is the exponential decay of the secret key rate as a function of channel loss, which effectively restricts fiber-based QKD to metropolitan scales. While Quantum Repeaters —which utilize entanglement swapping and quantum memory— could theoretically extend this range, they remain experimentally unfeasible for large-scale deployment. Currently, terrestrial long-distance networks rely on trusted nodes, often deployed using one of the most mature QKD protocols up to date, i.e. BB84. These intermediate relays perform classical decryption and re-encryption to forward keys, a process that precludes end-to-end quantum security and requires a high level of physical security for every node in the chain. To circumvent these limitations, satellite-based QKD has emerged as a superior alternative. Unlike optical fibers, where photons face constant attenuation (typically $0.2\text{ dB}/\mathrm{km}$), the majority of a satellite-to-ground link resides in a vacuum. Consequently, the effective path loss scales more favorably over longer distances, enabling the distribution of keys over intercontinental distances. By transitioning from terrestrial fiber backbones to orbital platforms, QKD can achieve higher key rates over long distances while potentially reducing the number of required trusted points in the network.

### State-of-the-art in QKD

The state-of-the-art in QKD is presented by introducing and reviewing a substantial selection of protocols. These are evaluated based on TRL and the secret bits per pulse metric relative to a given total loss (setup + channel losses) in dB. This provides insight for readers interested in identifying or estimate suitable QKD setups for deployment based on the analyzed metrics.

The considered QKD protocols and references commented in this section are only point-to-point QKD – they correspond to isolated QKD links. When multiple QKD nodes are integrated on a network, the secret QKD keys stored increase and decrease according to the generation and usage. To give an example, in a network made of trusted nodes, the secret keys stored in their corresponding databases can be increased or decreased dynamically in function of which node is the one forwarding the OTP key, as discussed in [76]. This section only discusses key generation capabilities of point-to-point links.

#### BB84 and considered performance metric

**Figure 1:**
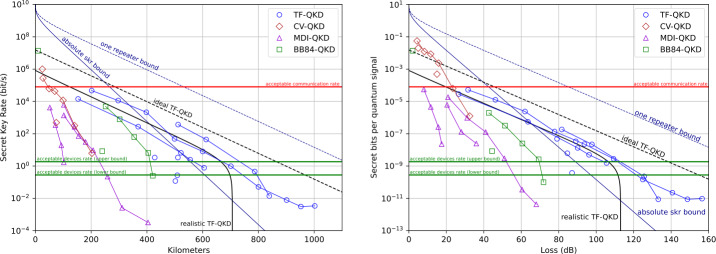
(Left) Simulated theoretical bounds with 0.16 dB loss per kilometer (non-horizontal solid lines) and experimental data from several QKD protocols plotted as secret key rate (bits per second) in function of distance (kilometers). Acceptable key rates for the “communication” and “device” rates are also plotted (horizontal solid lines); (Right) Same as the left figure but the secret key rate is expressed as secret bits per quantum signal in function of the loss in dB. A repetition rate of 1 GHz for the “communication” and “device” rates is assumed

Chronologically, QKD started with the seminal BB84 protocol, a DV PM scheme. In PM protocols, a sender prepares and transmits quantum signals through a lossy quantum channel to be detected and measured by a receiver. Such protocols are device-dependent and require rigorous device characterization of both the source and detectors to ensure that the physical implementation aligns with the mathematical models required to extract keys with Information-Theoretic Security (ITS). As the oldest protocol, BB84 has become one of the most understood and mature QKD protocols, making companies such as Toshiba and ID Quantique to deploy decoy states BB84, or the efficient version also known as the T12 protocol [49, 88], making the TRL of Decoy-States BB84 (and by extension T12 too) a 9 – agreeing with the TRL given in reference [35]. As for the performance of a QKD link in the finite-size regime, the values are estimated from Fig. 1, discussed later in this section: Decoy-States BB84 achieves a secret bit per pulse (SB/P) of the order of 10^−4^ for relatively low losses (20 dB), whereas 10^−5^ SB/P are achieved for relatively medium losses (40 dB) and no positive SB/P is attained for high losses (80 dB). The TRL and SB/P for the discussed QKD protocols are summarized in Table 2. In this summary, the BB84 label refers to both the Decoy-State BB84 and T12 protocol since they offer similar performance and security. See references [4, 6, 7, 20, 56, 74, 98, 102, 104] for reviews on QKD.

When evaluating SB/P, channel loss in dB is used as the primary metric because it provides a universal benchmark that is independent of the specific transmission medium. In terrestrial fiber-optic links, loss scales linearly with distance (often at $0.2\text{ dB}/\mathrm{km}$, meaning that losses of 20 dB, 40 dB, and 80 dB correspond to 100 km, 200 km, and 400 km, respectively). However, in satellite-to-ground links, a loss of 40–60 dB does not equate to the same physical limitations. Due to the vacuum of space and the geometry of satellite passes, such loss levels are reached over much larger physical distances (often thousands of kilometers) compared to fiber. Furthermore, satellite-based architectures often utilize trusted nodes or entanglement-based configurations (two honest parties interacting with a third non-trusted party), allowing for a secret key to be established between two ground stations whose physical separation far exceeds the limits of a direct fiber link with equivalent optical loss.

#### Discrete variable prepapre-and-measure protocols

Beyond BB84, several DV PM protocols have been developed to address specific environmental or security challenges. These include the Six-State Protocol (SSP) for higher noise tolerance [53], SARG04 [3] for robustness against photon-number-splitting attacks, and Reference-Frame-Independent (RFI) protocol [86], which could be a potential candidate for satellite-based applications. SSP allows to have a better information leakage bound, SARG04 is easier and cheaper to implement as decoy-states are not necessary and RFI offers an alternative where no active alignment is necessary. Despite these specialized advantages, Decoy-State BB84 remains the industry standard due to its superior performance in practical settings: see the SB/P in Table 2 where the values for SARG04, SSP and RFI are estimated given references [3, 96] and [86], respectively. The TRL of SSP, SARG04 and RFI are estimated to range between 5 and 7, given as they all have been implemented in the laboratory.

Additional DV PM schemes are Distributed-Phase-Reference protocols such as Differential Phase Shift (DPS), Coherent One Way (COW), and Round-Robin DPS (RRDPS), offering high-speed and cheaper alternatives by encoding information in the relative phase or timing of consecutive pulses. However, Decoy-States BB84 presents a better performance, notably for intermediate levels of loss (around 40 dB): see the SB/P in Table 2 where the values for DPS, RRDPS and COW are obtained from [22, 83, 101], respectively. The TRL of DPS and RRDPS are estimated to range between 5 and 7, given that they all have been implemented in the laboratory. The TRL of COW was claimed to be a 9 in reference [35] but a lower range is attributed in this work. Historically, the original COW protocol lacked a rigorous security proof against coherent attacks; while it was shown to be secure against specific attack classes [89], its security against general attacks (coherent attacks) remained unknown. Although a 2026 variant has finally established security against coherent attacks including finite-size effects [22]. Similarly, the security against coherent attacks with finite size effects has also been established in 2026 for DPS QKD protocols [83].

#### Device-independent protocols

Parallel to the development of BB84, the E91 QKD protocol [31] was independently introduced and has since become the foundation of DI-QKD. This represents the highest level of security available in quantum cryptography – a protocol where parties do not need to trust their hardware yet can maintain the certainty that the distilled keys are ITS [4, 6, 102]. Certain loopholes imposes constraints on how to implement DI-QKD which limit their current deployment even for low losses. In 2026, DI-QKD was implemented using a form of quantum memories that allowed to obtain a positive secret bit per pulse over 11 kilometers (or around 3.5 dB loss) [62]. Furthermore, they estimate a positive secret key rate for the asymptotic regime (the theoretical limit where an infinite number of signals are exchanged and statistical fluctuations are negligible) for distances up to 100 km (or around 32 dB loss for the idler signal). We anticipate that this rate would drop to zero once finite-size effects (the statistical penalties associated with processing a limited number of signals) are accounted for in the analysis.

Shortly after E91, its PM variant, the BBM92 protocol, was published. This protocol is source-independent as it relies on an untrusted source to distribute entangled qubits to remote honest parties, who then distill an ITS key via an authenticated classical channel. This is seen as a good candidate to implement satellite-based QKD, where the satellite is untrusted and distributes entanglement between two ground-stations [32] – see for example the Micius satellite work [100]. The TRL of BBM92 is estimated to range 5 to 7 as well given the entanglement-based TRL provided in [35] (TRL ranging 5-6) [35] and the deployment in a satellite. The SB/P is estimated from the implementation given in [24, 100].

This same architecture composed of two honest parties and an untrusted third party is found in (DV) MDI-QKD, the natural evolution of (DV) PM schemes. For a deeper exploration of DV MDI QKD schemes, we redirect the reader to the already mentioned QKD reviews [56, 74, 98]. The architecture of untrusted party is a natural candidate for star-like network topologies. The TRL of DV MDI QKD is estimated to range between 5 and 7 given their deployment in the field as the data points in Fig. 1 shows, and the estimated TRL (5-6) given in [35]. The SB/P is estimated from Fig. 1 which is discussed later in this section. Note that regarding satellite-based application, down-link is preferred over up-link thus BBM92 performs naturally better than MDI QKD.

An even further enhancement of MDI-QKD is the Twin-field (TF)-QKD protocol [7, 63] in which the third party acts virtually as a relay between honest parties, thus increasing the SB/P over high losses – see Fig. 1, in which values are based on the estimation given in Table 1. The TRL is estimated to be around 5-6 in [35]. TF-QKD is the foundation for long-distance QKD and could also be considered for satellite-based applications, resulting in a better performance than BBM92 under good conditions [32], despite having two up-links.

The most recent QKD protocols found in the literature are often based on TF-QKD, notably Mode-Pairing (MP)-QKD and Side-Channel-Secure (SCS)-QKD, which try to offer improved security guarantees or decrease the cost and complexity of deployment [7]. The TRL of MP and SCS-QKD range between 5-6 and 4-6, respectively, given their relatively recent appearance and homemade deployment in laboratories – see for example [29, 58, 103], where estimates of SB/P are also provided.

#### Continuous-variable QKD

Finally, while CV-QKD can also be MDI (e.g. [44]), and in theory DI too [4], the considered CV-protocols in this work follow the PM framework due to its superior maturity and performance. CV PM QKD schemes offer high performance in low-loss regimes as they involve distinct modulation and detection techniques. Furthermore, CV-QKD protocols have a relatively low cost compared to their DV-QKD counterparts, which could be preferable for the deployment on metropolitan distances. On the other side, DV-QKD achieves better secret key rates in medium and long distance, which cannot be achieved by CV-QKD, in exchange of an increase in cost [5]. The SB/P for CV-QKD is estimated from Fig. 1 which is discussed later in this section. For a deeper exploration of CV PM schemes, we redirect the reader to specialized reviews such as [20, 104].

All the information commented in this section is summarized in Table 2.

#### Performance evaluation

Common QKD protocols that have been deployed are decoy-states BB84 (or T12), BBM92, MDI and TF-QKD for the DV side and Gaussian Modulation (GM) CV-QKD or Discrete Modulation (DM) CV-QKD for the CV side. Figure 1 describes how the SB/P evolves in function of the loss (or distance assuming an attenuation of 0.2 dB/km), using data obtained from actual implementations. This is useful to estimate the order of magnitude of the SB/P in the scenarios that we present in Sect. 5.

The left and right panels of Fig. 1 present the simulated bounds of the secret key rate as a function of channel loss and distance (fiber link), taken from [63, 73, 75]. These plots include the one-repeater bound, the fundamental secret key capacity, commonly known as the PLOB bound, and the ideal and realistic cases for TF-QKD. The PLOB bound allows to visualize how the secret key rate (or equivalently the SB/P) evolves in function of the loss levels given for any point-to-point quantum channel. It scales linearly with the channel transmittance, or $10^{\frac{-\text{loss in dB}}{10}}$. As shown in Fig. 1, standard protocols such as BB84, MDI, and CV-QKD are strictly bounded by this limit. In 2018, the introduction of TF-QKD [63] overcame this restriction. By utilizing a central interference midpoint, TF-QKD achieves a repeater-like scaling that proportional to the square root of the transmittance, or $10^{\frac{-\text{loss in dB}}{20}}$. This allows the protocol to significantly surpass the PLOB bound at high loss levels. The figure also includes a theoretical bound for TF-QKD considering realistic devices, along with variants like MP and SCS-QKD which share this improved scaling. Note that these theoretical bounds are plotted with 0.16 dB loss per kilometer and 1 GHz repetition rate while converting the x-axis and y-axis into kilometers and secret key rate, respectively (left side of Fig. 1).

Experimental data comparing point-to-point QKD links of various QKD protocols (BB84, MDI, CV, and TF) sourced from [60, 93, 98] and the references therein are also plotted. While most reported testbeds utilize fiber-based links, a small subset involves free-space or satellite channels. To avoid ambiguity between distance in fiber and free-space, Fig. 1 (right) presents the SB/P as a function of channel loss (dB). This provides a more objective comparison across different transmission media. Although many studies report the secret key rate (SKR) relative to distance (km), the total quantum channel and device losses are extracted from the respective references to ensure a consistent metric. To convert between SKR and BP/S, only the repetition rate of the quantum source is required.

The experimental data from Fig. 1 agrees with the first paragraphs of this section: CV-QKD is ideal for short distances (low noise) whereas MDI-QKD and BB84 become a better candidate for increased distances. BB84 is a choice of preference for its low complexity of deployment, cost and comparable performance to MDI-QKD. For long-distance, TF-QKD is one of the few options. The other option is to adapt recently developed variants such as MP or SCS-QKD protocols.

Acceptable key rate bounds per user for applications have been plotted following the values discussed in the use cases of Sect. 5.2, where the QKD setups are constrained to operate 3 hours per day. This limit represents the fact that most satellite-based QKD links operate only during the night and can see their availability further reduced by meteorological conditions. The “acceptable communication rate” represents the required bit rate to obtain enough bits every day to fully encrypt with one-time pad (OTP) a communication channel per user – 10 kilobits per second are required, which corresponds to 80 kilobits per second generated throughout the daily QKD operational window. The lower and upper bound of “acceptable device rates” corresponds to the required bit rate to obtain enough bits every day to run devices per user - around 3 to 20 kilobits per day are required, which corresponds around 0.28 to 1.85 bits per second generated the daily QKD operational window. The bounds in the right side of Fig. 1 are computed by assuming a repetition rate of 1 GHz. It is worth mentioning that if OTP encryption is replaced by symmetric cryptography such as Advanced Encryption Standard (AES) encryption then the acceptable key rate would decrease substantially since one key can be used to encrypt multiple data packages.

As Fig. 1 shows, QKD is able to go past the “acceptable communication rate” for short distances, achievable by CV-QKD and BB84 under distances of 200 kilometers (or under 40 dB loss). As for the “acceptable device rates”, the distance can be extended up to around 800 kilometers (or 120 dB loss) – CV, BB84, MDI and TF-QKD are suitable candidates for short distances (less than 200 kilometers or 40 dB loss), BB84 and TF-QKD are suitable for medium distances (between 200 and 400 kilometers or 40 dB and 70 dB loss) and TF-QKD is the only candidate for longer distances, up to around 800 kilometers (or 120 dB loss).

For terrestrial applications, fiber-link and free-space applications typically rely on Decoy-States BB84 for its high TRL. Regarding communications between trusted satellite and ground-station, Decoy-States BB84 is also a viable option when the satellite is the one acting as the source due to acceptable losses (around 40-50 dB loss) [32, 70].

For untrusted satellites applications, source-independent QKD is a good candidate, notably the BBM92 protocol, where the satellite distributes entangled qubits between honest parties. This is due to the lower losses that light presents when the path from the satellite to the ground-stations is down-link compared to the up-link direction. For BBM92, the orders of magnitude of secret bit per quantum signal provided in Table 2 are obtained through the Micius satellite work [100] (around 0.12 bps for 1120 kilometers or around $2.03\times 10^{-8}$ secret bits per quantum signal for losses that go from 33 to 42, for a repetition rate of 5.9 MHz) and the realistic simulation from [24] (around 10 bps are extrapolated for around 20 dB loss with a repetition rate of 1.5 MHz, giving around 10^−5^ secret bits per quantum pulse). While the landmark Micius satellite mission reported a rate of 0.12 bps under extreme losses, this figure represents a boundary-pushing record for long-distance communication rather than a baseline for standard operations. Conversely, the extrapolated 20 bps from the realistic simulation illustrates that while experimental extremes currently sit at the lower edge of the “acceptable device rates” threshold (0.28 to 1.85 bps), BBM92 is technically capable of exceeding the target threshold under standard operational configurations.

BBM92 is expected to scale as MDI-QKD in terms of secret key rates in function of loss since the qubits from both arms have to survive the trip from the source to honest parties to distill a secret key. As mentioned in a previous paragraph, other candidates to consider to perform satellite-based QKD with an untrusted middle node is TF-QKD, notably their variants such as MP where phase locking is not a problem. TF-QKD is shown to perform better than BBM92 in simulations despite having two up-links [32], whereas MP could be used to remove the phase locking complexity that TF requires [7].

When it comes to device-independent QKD, its performances are poor even for short distances, thus making it unpractical for deployment in the near future [4]. However, significant advances keep the technology forward every year – see the corresponding 10 km milestone achieved in 2026 [62].

Finally, note that the SB/P can be improved at higher losses with post-processing techniques that are not taken into account in this work. An example is advantage distillation, which allows to extract a secret key with higher quantum bit error rates than the allowed threshold [45].

This section has presented several QKD protocols and their corresponding performance in terms of loss. Decoy-States BB84 is the preferred choice for industry due to its good trade off between complexity implementation, cost and performance. However the emergence of protocols that present specific advantages over BB84 such as RFI (Reference independent property) and COW (less complex and cheaper to deploy) are possible since they have been shown to be resistant to coherent attacks and analyzes with finite size effects in 2026. As for untrusted nodes, TF-QKD and their variants are of high interest given the better performance in the high loss regime. As for trusted satellite-based applications, Decoy-State BB84 remains a good candidate at the time of writing. As for untrusted applications, TF-QKD and their variants are relevant in terrestrial and satellite-based protocols. However, BBM92 remains the norm for untrusted satellite-based applications; TF-QKD and their variants are expected to emerge however, given the potential higher performance.

### Current deployment: overview of QKD for dual-use

The global deployment of QKD has evolved into three distinct architectural and strategic approaches: terrestrial fiber backbones, satellite-based intercontinental links, and divergent national security paradigms. This section presents relevant QKD networks that have been deployed around the world in a non-exhaustive way, and does not discuss whether the implemented protocols are secure against side-channels or not.

Terrestrial deployments focus on connecting high-density governmental, financial, and industrial hubs. As for established networks, China presents a worldwide lead with the interconnected China Quantum Communication Network (CN-QCN) and the Beijing-Shanghai Backbone Network (BSBN), a 12,000 km network featuring 145 nodes (including 104 relay nodes) supporting over 800 users [25]. Similarly, Russia has deployed a 7000 km network [85], and South Korea maintains an 800 km link securing 48 government departments [48]. Additionally, Singapore has been developing its quantum-safe communications infrastructure since 2022 [50]. Regarding the European Union, deployment of national Quantum Communication Infrastructures through projects such as PIONIER-Q (Poland) [72] and RoNaQCI (Romania) [82], currently the largest such networks in the EU, alongside with smaller networks such as FranceQCI (France) [71] and QUID (Italy) [26], are being made under the EuroQCI initiative [34]. These national segments, funded by the Digital Europe Programme (DEP), serve as the foundation for a future cross-border backbone to be interconnected through the Connecting Europe Facility (CEF). In the UK, the London Quantum Secured Metro Network [61] serves as a commercial trial for telecommunications, while a 410 km link connects Bristol and Cambridge [99]. Industrial integration has been also a point of interest, for example, Japan is integrating QKD into the Innovative Optical and Wireless Network (IOWN) [87], while India has demonstrated 500 km links through collaborations between startups and the military [68].

To overcome the distance limitations of fiber, satellite-based QKD is being used to create global quantum links. China’s backbone includes six nodes equipped with satellite ground stations. This has enabled functional intercontinental links with Russia [85] and South Africa [57]. Singapore and the UK collaborated on the SpeQtre satellite (launched in 2025) to test space-to-ground performance [91]. Singapore is also deploying its own satellite-based link [65], while Canada has scheduled a demonstration for 2026 [51].

The deployment of QKD is often dictated by national security positions regarding dual-use technology. China’s massive QKD infrastructure is a direct application of its Military-Civil Fusion strategy, which blends civilian infrastructure with military requirements [90]. This is evidenced by the use of the BSBN by financial institutions as early as 2017 while simultaneously developing hybrid QKD-PQC systems for state security [2, 97]. The European Commission similarly argues that the dual-use potential for civilian and military areas is a primary driver for the EuroQCI investment [33, 34]. In contrast, the USA government (via the NSA and DoD) does not back QKD, recommending Post-Quantum Cryptography (PQC) instead. This has shifted US QKD development to the private sector, such as the Quantum Corridor testbed [78]. A similar trend is seen in Australia [1].

To summarize, the QKD technology is mainly used by institutions that have the means to buy, implement and maintain such as finance, health, telecommunications and government institutions. QKD can potentially be used by military institutions where trusted nodes may play a bigger role.

While QKD theoretically provides ITS, practical non-DI implementations remain susceptible to implementation defects that could result in security breaches. Consequently, the hybridization of QKD with other quantum-safe primitives, such as Post-Quantum Cryptography (PQC), is currently a major focus of research.

### QKD integration within hybrid protocols

At the heart of hybridization lies the theoretical notion of universal composability. For example, QKD protocols that possess a security proof based on entanglement purification are proven to be universally composable [11]. This composability allows QKD to be seamlessly integrated with classical primitives like PQC.

Despite the strong theoretic background, any QKD implementation that deviates from the corresponding security proof, for example because of imperfect devices, has no guarantee to be secure anymore. Furthermore, said security proofs do not take into account side-channel attacks, vulnerabilities born from the hardware that are exploited by any classical adversary to obtain side information, rendering the final key with no guarantee to be secure again [6]. A case example of QKD implementation that was found out to be insecure later is the Decoy-States BB84 run by the Micius satellite [59]. In a nutshell, engineering problems led to a bad implementation of decoys state which voids the ITS guarantee [67].

The counter-measure to make QKD secure as promised is to identify all of the possible side-channel attacks and provide hardware (or software) solutions to every single one of them – whether it is possible to protect every single piece of hardware or not remains a subject of active research [5]. DI- or MDI-QKD protocols also play a relevant role as discussed in Sect. 3.1. A QKD implementation that presents no exploitable side-channels and whose devices are characterized (if required), could be given a certification stating its security, provided by recognized standardization organizations that are involved with QKD such as the European Telecommunications Standards Institute (ETSI) [37] or the International Organization for Standardization (ISO) [52].

Despite the lack of security certification, deployment of QKD setups has not ceased and keeps expanding in these recent years. Once certification of QKD links becomes a worldwide recognized standardization tool, one can certificate the already deployed QKD setups easily.

The hybridization of QKD and PQC is commented in the next Sect. 4.

## Hybrid authenticated key exchange and related protocols

With QKD introduced, the present section presents how QKD can be hybridized with PQC and what benefits can this hybrid approach bring. While cryptography can serve a very wide variety of purposes, we restrict to the most essential feature: authenticated key-exchange protocols. Before discussing specific HAKE protocols, we provide an overview of the current and emerging PQC algorithms standardized by NIST.

### Standardized PQC algorithms

Currently, the primary NIST-standardized PQC algorithm for general-purpose key exchange is the Key Encapsulation Mechanism (KEM) ML-KEM (FIPS 203). While it is the only lattice-based KEM currently finalized, HQC (slated to become FIPS 207) was selected in 2025 to provide a code-based alternative, with a final standard expected by 2027. For authentication, NIST has standardized several digital signature algorithms (DSAs): the stateless ML-DSA (FIPS 204) and SLH-DSA (FIPS 205 and Special Publication 800-230), as well as the stateful hash-based schemes such as eXtended Merkle Signature Scheme (XMSS) and Leighton-Micali Signature (LMS), both standardized under the Special Publication (SP) 800-208. Furthermore, a third stateless signature standard, FN-DSA (Falcon), is in the final stages of development as FIPS 206 to address applications requiring smaller signature sizes. The mentioned PQC algorithms are summarized in Table 3. Note that it can be acceptable to use other alternatives such as FrodoKEM [43], which is backed by ANSSI, BSI and several other European agencies.

Apart from constraint applications such as Internet of Things (IoT), Domain Name System Security Extensions (DNSSEC) or underwater communications, PQC rarely poses technical difficulties within authenticated key exchange (AKE) protocols, even when using satellite communications. Indeed, Low Earth Orbit (LEO) constellations providing internet connectivity can boast down-link speeds up to several gigabits per second (Gbps) [28]. Even considering more challenging scenarios such as inter-satellite communications or even Earth-Moon communications [8], it is realistic to assume data rates of 10 megabits per second (Mbps) and higher. All the HAKE protocols in the literature consist in exchanging a few copies (less than four) of the following objects: public keys, ciphertexts and signatures (integrity tags and headers are neglected as their bit sizes are several orders of magnitude smaller). Depending on the PQC algorithms that are used, these objects have sizes ranging from several kilobytes (KB), typically for structured lattices, to 35 KB for an SLH-DSA signature. Therefore, we consider in what is following that we have more than enough link budget to accommodate for the use of any standardized PQC primitives.

This is why we decide not to discuss the choice of a specific cryptographic algorithm, as all the ones mentioned in Table 3 fit our needs, and even more of them could be considered. Such an approach is also dictated by crypto-agility, a standard good practice for PQC. This means that protocols should remain agnostic of the choice of the underlying cryptographic primitives so that they could easily be replaced by another one, should they be proven insecure in the future.

### Combined hybrid protocols

Inspired by the definition of classical and post-quantum hybrid protocols such as [16], a natural design is to integrate QKD within an Authenticated Key Exchange (AKE) protocol by combining the key derivation with authentication [69]. This way, the classical channel is authenticated either via post-quantum primitives such as signatures [18, 21, 36, 41], Key Encapsulation Mechanisms (KEMs) [10, 47], via PSKs [21, 30, 41], or via an ITS MAC such as the Carter-Wegman construction [94]. Because the resulting session key is derived from key material coming from both post-quantum and quantum cryptography, these protocols are referred to as “Combined Hybrid Protocols”.

Hybrid protocols that rely on post-quantum primitives to authenticate the key exchange fail to provide full information-theoretic security as a computationally unbounded attacker trivially compromises the authentication step and impersonates honest parties, thus recovering the session key. However, if the attacker fails to do so during the execution of the protocol, the only way to get access to the session key is to break both the post-quantum and quantum cryptography. Under the usual assumptions made on QKD, this means that even an unbounded attacker cannot retrieve the session key once the protocol has successfully terminated: QKD provides *everlasting security*.

Note that this summary makes it look very straightforward that such HAKE protocols reach everlasting security but it is not the case, see [47] for a detailed analysis of security guarantees offered by various designs and the many pitfalls encountered when proving security.

The cornerstone within hybrid protocols is the combination of the KEM and QKD secrets. The most natural approach would be to simply XOR the keys but this would only defeat a passive attacker and fail to provide Indistinguishability under Chosen Ciphertext Attack (IND-CCA) security when an active attacker is considered. The current standard is to use the XOR-then-MAC (XtM) paradigm introduced in [17], which is so far the only construction to provide IND-CCA security without making any computational assumption. More precisely, this consists on performing quantum-resilient MACs (sometimes noted as one-time MACs) with part of the keys to combine, and output the XOR of the remainder of the keys as the session key in the case that the MAC tags are verified successfully. It is applied to combine KEM and QKD secrets in [47], where the HAKE protocol is shown to be ITS in the case scenario where the QKD secret remains secure. The XtM paradigm is crucial when aiming for full ITS but when everlasting security is enough, it is acceptable to use a computational MAC (e.g., HMAC or rely on the HKDF paradigm [55]).

One may wonder why [47] even bothers with PQC since they want to achieve information-theoretic security, a goal which PQC cannot attain. The reason is three-fold: providing defense-in-depth in case QKD is affected by side-channel attacks, providing (computational) end-to-end security to avoid eavesdropping by relay nodes within the quantum network, and ensuring authentication in the case scenario QKD nodes have no other way to authenticate, reducing their security from ITS to everlasting security. Indeed, a significant assumption made in [47] is that only the honest parties have access to the QKD keys. In practice, either this assumption is ensured by operational measures or we have to use computational cryptographic assumptions to provide authentication for the QKD channel in order to ensure that the right parties have the right QKD keys. Of course, if we use the latter option we cannot hope for information-theoretic security.

Therefore, we consider HAKE and related protocols from Table 1 that base the security of their authentication mechanism on PQC. All protocols achieve at least computational (defense-in-depth) security in the HAKE or related frameworks.^3^ Noteworthy, VMuckle-ES [36] and ITS AKE [47] provide at least everlasting security where the shared key achieves even ITS after the protocol run.

All protocols from Table 1 are agnostic to the actual instantiations of the QKD and PQC components, except for NBB AKE [38] which uses a non-black-box-QKD security model and is, hence, limited in terms of choosing a non-BB84 QKD protocol for a dedicated use case.

Besides security guarantees, Muckle# [10] and ITS AKE [47] have been somewhat optimized for PQC as those provide authentication using post-quantum KEMs instead of post-quantum signatures, based on the observation that post-quantum KEMs are more efficient than post-quantum signatures. Another important feature for real-life deployment is versatility and crypto-agility, where Muckle++ [41], VMuckle [21], and VMuckle-ES [36] stand out (since those protocols have the additional possibilities of including pre-shared authentication secrets, e.g., as a fallback mechanism). Moreover, VMuckle-ES [36] is currently in stable draft form for a Technical Specification (TS) 104,146 V0.0.4 at ETSI.

Note that, as mentioned in [47], it is paramount to also associate key identifiers to QKD key material and to include these identifiers in the protocol steps in order to avoid mismatch attacks. So far, only ITS AKE [47] and VMuckle-ES [36] deploy QKD key identifiers.

All those mentioned types of QKD-PQC hybridization protocols from Table 1 with the features discussed above seem to be the most readily deployable in the near future as those can make use of already existing high-TRL solutions for both post-quantum KEMs/signatures and QKD. However, as pointed out earlier, quantum cryptography goes far beyond QKD and hence this design paradigm may eventually become too rigid and lack more advanced functionalities as quantum cryptography gains in maturity and versatility.

This brings us to another way of hybridizing which also achieves everlasting security but for which the computational assumption is of a different nature and applies to the quantum channel.

### Hybrid protocols perspective

In order to avoid relying on computational assumption, one can also make security assumptions on the quantum storage capabilities of an attacker. For instance, the bounded quantum storage model of [27] proposes a protocol that is secure as long as an adversary has access to a limited quantum memory. Another option is to make assumption not on the size of the memory but on its coherence time, i.e. the time after which a qubit loses its quantum properties (e.g. entanglement and superposition) due to interactions with the environment. An example of this is the quantum data locking (QDL) paradigm [64], where the quantum memory is assumed to completely decohere after a finite time. A more refined model is the noisy storage model [54, 95], where the memory does not completely decoherence but becomes noisy after some time.

The protocol introduced in [66] works in the noisy storage model but also adds computational assumptions ensuring that an adversary cannot perform some operations before its quantum memory becomes noisy. The main benefit of this approach is that it allows to send $O\left (\frac{\sqrt{n}}{\log n}\right )$ photon per use of the quantum channel, while previous solutions would only allow to use one single photon. This represents a significant increase in the key rates and allows the author to propose more flexible use-cases (e.g. multicast) as well as a semi-device independent variant whose goal is to adapt to untrusted devices.

While these protocols will certainly play a major role in the future thanks to the performance they offer, it seems currently quite challenging to design and mass produce hardware that would make them a reality. Therefore, we do not discuss these protocols in what follows and focus on combiner-based protocols.

## Dual use and international policies

Over the past years, national security organizations have being quite unanimous in dismissing QKD. While acknowledging that it was important to pursue research effort in the field, both American and European agencies pointed out the lack of maturity of QKD, its vulnerability to denial of service attack, its reliance on hardware quality or honesty and its practical limitations.

While we believe that this harsh position was partly adopted to avoid some organizations seeing QKD as a replacement to PQC, the result is that no certification criteria have been issued regarding QKD, which may in turn result in the adoption of poorly secured solutions. Our position is that any technology should not be judged by its strengths or its limitations, but should rather be studied on a case-by-case basis, so that adoption could be approved in relevant cases and disallowed otherwise.

In this section, we propose a few dual-use cases for which QKD could be of interest, and justify why the limitations of QKD may be overlooked in these cases. For these use-cases, we made assumptions on the QKD availability and propose targeted key rates based on Sect. 5.2.

From this, we deduce figures of merit on the size limits of the network (both in geographical distance and number of users). These assumptions are arbitrary but our goal is that our figures of merit can serve engineers to make preliminary assessments when dimensioning a project: since most of the parameters have a linear impact, it is straightforward to adjust our results to any practical use case. For instance, in a scenario when QKD is expected to be operational twice more often, the number of users can be doubled or the acceptable key rate can be divided by two. Conversely, if the intended number of users is larger (resp. smaller) by a factor *k* compared to our figures, then the acceptable key rate will have to be multiplied (resp. divided) by *k*.

### Use cases

During military operations, various types of radio frequency (RF) networks are deployed, from local networks directly used on the field (typically HF/VHF/UHF links with ranges from tens to hundreds of kilometers) to long-range communications between the strategic command (e.g. the Ministry of Defense premises) and a military vessel or a forward operating base. These communications rely on SATCOM since the distance to cover can be up to (tens of) thousands of kilometers. For such application, trusting the hardware is not a very strong assumption since the communicating devices are located in trusted areas and operated by appropriately cleared personnel. Also, since the command nodes are not located directly in a contested environment, it is safe to assume that an attacker has a limited capacity in terms of denial of service (e.g. blinding or jamming).

Further use cases under which similar assumptions could be made include local ground-communication networks between strategic actors (ministries, industry and military commands) or inter-satellite communications, for instance within a SATCOM constellation or between distinct constellations (e.g. between GEO and LEO satellites). On such sensitive networks, parties are, in some sort, partly authenticated by the fact that they have access to terminals located in secure facilities (e.g. personnel must hold an appropriate clearance and access is secured by badges, codes and biometry). While this is far from the guarantees of cryptographic authentication, we believe that it is sufficient to ensure that parties will not attempt DoS attacks. Depending on the level of trust, it might also make it acceptable to use the protocol from [47] if such measures are considered enough to guarantee honest use of the QKD channel. However, a key factor to consider is whether information must be segregated on a need-to-know basis (e.g., level of clearance or nationality), which would necessitate the rigorous guarantees of cryptographic QKD key authentication.

In the civilian world, the unitary cost of QKD devices is still too high at the moment to consider large scale applications. However, the banking industry is interested in this technology for specific endpoints, for instance to secure communications between Central Banks, between banks themselves for Central Counterparty Clearing or between banks and certain customers that stand out by their size or the sensitivity of their business. Short, medium and long distance QKD links can be implemented accordingly with CV, Decoy-States BB84 and TF-QKD protocols, respectively. While SI- or MDI-QKD (including TF, MP and their variants) have great potential for star-network topologies, where multiple nodes can connect to a source or detectors, respectively, their TRL or low performance in relatively high losses makes the technology to require further investment to make its deployment viable. Decoy-States BB84 is the preferred protocol to be deployed given the high TRL and performance. Note that further operational measures are to be taken to mitigate the risk of DoS attacks (e.g. terminals located in secure areas or diplomatic measures to ensure that satellites are not harmed in peace time).

An interesting distinction between military and civilian use cases for satellite-based QKD resides in the choice of the protocol. Since the military will necessarily have to rely on dedicated military-purpose satellites for obvious security reasons, there is no significant issue with trusting the device within the satellite itself, hence a protocol such as Decoy-States BB84, where the satellite acts as the source, is both suitable and convenient, as mentioned in Sect. 3.2.

For civilian purposes, however, it is most likely that users have no reason to trust the satellite manufacturers, especially given how long the chain of subcontracting can be in such projects. One could also envision a future economic model of QKD where there is a pool of satellites launched by many countries, to which users occasionally buy service time. In this case, it becomes all the more important to use protocols that allow to have an untrusted third party such as source-independent-based, measurement-device-independent-based, twin-field or the more recent mode-pairing protocols. The current state of art regarding satellites is to lean into BBM92, a source-independent protocol where the satellite acts as the source for honest parties.

This kind of asset sharing could also extend to the military world, when considering alliances or coalitions. Note that even if the nation operating the satellite cannot eavesdrop, it can still decide to turn its satellite off, thus effectively performing a denial of service.

### Acceptable key rates and network size

Once hybrid protocols are used to negotiate key material, different options are possible. The first and most secure option is to use the key material to ensure information-theoretic (or everlasting) security to every data exchanged through the channel, in which case the algorithms used for encryption much provide the same level of guarantee. This is typically the case for highly sensitive information such as communications between political leaders and deployed forces, as described above.

For less sensitive applications, however, we may want to have key material negotiated through a protocol offering ITS or everlasting security, while still being willing to use such key material within classical symmetric algorithms. This might seem incongruous to do so after making the effort of going through QKD but it makes sense for applications in which the lifetime of the negotiated secret key is short, and the threat of compromise inexistent. This is often the case for military exchanges on the battle field: either the tactical communications need not be secret for a long time (most orders or battle plans are no longer secret when the operation ends), or the key is solely used for authentication purposed (e.g. in IFF systems whose goal is to distinguish allied from hostile forces), or keys used to protect signal instead of data (e.g. frequency hopping). This also applies to banking transactions where integrity and authenticity may be more important than long-term secrecy.

Another benefit of this approach is that everlasting secure keys can be stored even for a very long period of time without posing a security risk. This is particularly interesting for keys placed in equipments with limited accessibility (e.g. remote stations beyond the polar circle, space missions, military submarines and surface vessels).

This gives us two different cases with completely different consumption of keying material. In the first case, the key rate essentially dictates the communication rate. Assuming that the exchange must ensure voice and text communication, a minimum rate of a 10 kilobits per second (kbps) is advised, based on GSM standards. Assuming that QKD can only be achieved during 3 hours per day (e.g. because of satellite visibility or weather conditions) and that one-time pad with appropriate integrity checks is chosen as the data-encryption scheme, we must target key rates of about 80 kbps to ensure a secure communication all day long. This value must of course be multiplied by the intended number of endpoints to be deployed at the same time. The rates presented in Sect. 3.2 show that even a dozen of users would be hard to accommodate when using MDI or CV-QKD for distances around 25 kilometers (i.e. loss around 10 dB), which leads us to consider that aiming for ITS security is not realistic with the current technology, unless very specific use cases are considered.

Let us now consider a network of a hundred users where all users have a QKD link with each other, that they use to negotiate daily symmetric keys (they do not apply one-time pad as above). The key material required amounts to $256\times \frac{100(100-1)}{2}$ (one key per link and per day), which amounts to 1.3 Mb of keying material. Assuming once again that QKD is only operational 3 hours per day, it requires a key rate of 117 bps. According to Fig. 1, using BB84, the QKD links can range over a distance up to approximately 350 km, while using MDI or BBM92 results in a maximal distance below 200 km.

Even with ten thousand users, the above network becomes irrelevant in practice because the size of keying material is increased by a factor 10^4^, resulting in a key rate of approximately 10^6^, so far only practical for very small distances (low loss regime).

One may argue that some of our applications result in networks with specific topologies: strategic information, for instance, is exchanged to and from central command but need not be shared directly from one node to another, potentially achieved with MDI or BBM92 protocols. In that case, the number of symmetric keys to be generated is equal to the number of users (minus one) instead of being quadratic. Using this topology and assuming key rates between 100 and 1000 bps, the network has the potential to accommodate about four thousand to forty thousands users.

## Conclusion

We sum up the use cases discussed in this paper, and provide examples of suitable options as well as feasible network configurations based on the current state of the art (see Table 4).

**Table 4:** Features and suggested protocol for each use case

Use case	Data to protect	Network	Suitable protocol security
Ground network	Full traffic	25 km, 10 nodes	ITS or Everlasting using BB84
Defense use	Symmetric keys	350 km, 100 users	Computational or Everlasting using BB84
Civilian use	Symmetric keys	200 km, 100 users	Computational or Everlasting using BBM92
Star-shaped	Symmetric keys	350 km, 10,000 users	Computational or Everlasting using BB84

For ITS or Everlasting Secure protocols, ITS AKE and VMuckle-ES are both suitable. For computational secure protocol and no efficiency considerations, Muckle, Muckle+, Muckle++, and VMuckle are suitable; for computational secure and efficiency considerations, Muckle# is suitable.

The rationale for these limits and suggestions have been discussed in Sect. 5 but we provide a quick summary:

- Use of the ITS AKE [47] is only advised when targeting ITS security of traffic.
- BB84 is a good candidate when devices are trusted (defense case) and BBM92 is a suitable choice for civilian satellite-based QKD.
- When using Muckle variants, Muckle# [10] is a suitable choice for its performance, whereas VMuckle is a good candidate for flexibility [21]. When ITS security is required, VMuckle-ES [36] is a good candidate along with the ITS AKE protocol [47]. Else, the rest of the Muckle protocols [18, 30, 41] may be considered.
- Implementation of the HAKE protocol should be crypto-agile (allowing seamless change of cryptographic algorithm) and the underlying KEMs and signatures should come from an approved list (i.e. NIST standards or national recommendations).

## Data Availability

No datasets were generated or analysed during the current study.

## Footnotes

1 https://csrc.nist.gov/projects/post-quantum-cryptography#pqc-standards.

2 Note that the security model and protocol from [38] are tightly related to the specific QKD protocol BB84.

3 Note that in the current pre-print version of [47], it is mentioned that the security analysis of Muckle# [10] is incomplete due to unclear notions of malicious acceptance and cleanness. This, however, referred only to an early pre-print of Muckle#. Particularly, in the final published version of Muckle# [10], malicious acceptance is clearly stated as an honest session accepting the final MAC without an honest partner and there is an exhaustive list of allowed queries in the paragraph *Adversarial interaction*.
